# Effect of Cooling Rate on α Variant Selection and Microstructure Evolution in TB17 Titanium Alloy

**DOI:** 10.3390/ma17205010

**Published:** 2024-10-13

**Authors:** Guoqiang Shang, Xueping Gan, Xinnan Wang, Jinyang Ge, Chao Li, Zhishou Zhu, Xiaoyong Zhang, Kechao Zhou

**Affiliations:** 1 State Key Laboratory of Powder Metallurgy, Central South University, Changsha 410083, China; shanggq1984@126.com (G.S.); 203301071@csu.edu.cn (J.G.); zhoukechao@csu.edu.cn (K.Z.); 2 Key Laboratory of Advanced Titanium Alloys, AECC Beijing Institute of Aeronautical Materials, Beijing 100095, China; nansmily@126.com (X.W.); zhuzzs@126.com (Z.Z.); 3 Hunan Engineering Technology Research Center in Special Titanium Alloys for High-end Equipment, Hunan Goldsky Titanium Industry Technology Co., Ltd., Changde 415001, China; lc@xtjtty.com

**Keywords:** TB17 titanium alloy, microstructure evolution, variant selection

## Abstract

The α variant selection and microstructure evolution in a new metastable β titanium alloy TB17 were studied in depth by DTA, microhardness, XRD, SEM, and EBSD characterization methods. Under the rapid cooling rate conditions (150 °C/min–400 °C/min), only a very small amount of granular α_WM_ (α Widmanstatten precipitates within the grains) precipitated within the grains. The secondary α phase precipitated in the alloy changed from granular to fine needle-like at moderate cooling rates (15 °C/min–20 °C/min). When continuing to slow down the cooling rates (10 °C/min and 1 °C/min), the α_GB_ (α precipitates along the β grain boundaries), α_WGB_ (α Widmanstatten precipitates that developed from β grain boundaries or α_GB_) and α_WM_ grew rapidly. Moreover, the continuous cooling transformation (CCT) diagram illustrated the effect of cooling rate on the β/α phase transition. EBSD analysis revealed that the variants selection of α near the original β grain boundary is mainly divided into three categories. (i) The double-BOR (Burgers orientation relationship) α_WGB_ colonies within neighboring β grains grow in different directions but have the same crystallographic orientation. (ii) The double-BOR α_WGB_ colonies within neighboring β grains have different growth directions and different crystallographic orientations. (iii) The double-BOR α_WGB_ colonies within the same grain have the same growth direction, but different crystallographic directions. And these double-BOR α_WGB_ colonies correspond to two variants of the given {0001}α//{110}β.

## 1. Introduction

Metastable β-type titanium alloy has become the key direction of the development and application research of new titanium alloy, due to its good plasticity, high strength, high fracture toughness [1,2,3,4,5]. Among them, using as a new type of military advanced material, the ultra-high strength and toughness titanium alloy has been focused on and given priority to development at home and abroad [6,7,8]. Recently, a new metastable β titanium alloy with independent intellectual property rights for aircraft structure, TB17 alloy, has been developed by the Beijing Institute of Aeronautical Materials (BIAM). By adjusting the heat treatment process, the matching of ultra-high strength (Rm ≥ 1350 MPa) -plasticity (A ≥ 6%)and -toughness (K_IC_ ≥ 50 MPa·m^1/2^) can be realized [9,10,11], which can be applied to the load-bearing components or load-bearing bolt fasteners with high weight loss, high load and high life requirements of the new generation of aircraft.

Generally, the mechanical properties of alloys are closely related to the changes in microstructure. The microstructure of metastable β-type titanium alloy is determined by its processing and heat treatment state [12]. The mechanical properties of alloys can be modified over a wide range by changing the heat treatment process, such as solid solution temperature, cooling rate, and aging process, to achieve the matching of different strength, plasticity, and toughness levels [13,14,15]. Among them, the cooling process after solution treatment is one of the most important links of metastable β-type titanium alloy [16,17]. With the different cooling rates of solution treatment, β phase may directly precipitate α phase, or maybe decompose into the intermediate transition phase such as the ω phase [18], β′ phase [19], and α″ phase [20]. Therefore, in the case of a certain alloy composition, the phase transformation of titanium alloy during the cooling process mainly depends on the cooling rate. It determines the morphology, distribution, size, and type of phases in the titanium alloy structure at room temperature. For example, Angelier [21] has investigated the phase transition process of β-CEZ during continuous cooling after β-solution treatment. The results indicate that different cooling rates lead to the α/β structure showing two different morphologies: the basket structure at high cooling rates and the colony structure at low cooling rates. At intermediate cooling rates, both morphologies coexist. Many researchers have investigated the precipitation order and phase transition kinetics of different kinds of α precipitates, i.e., α_GB_, α_WGB_, and α_WM_. However, it is not fully clarified how the different types of α affect the microstructure. In addition, the α variant selection during the cooling process is also an important component of the microstructural changes in alloys. Xu et al. [22] investigated the precipitation behavior and evolution mechanism of grain boundary α (αGB) and α side plates (αSP) of TB-17 titanium alloy, the results showed that the αGB can change the morphology of β grain boundary, and the deformation degree of αGB increases with the increasing λ (The λ, which denotes the minimum value of misorientations between 12 α variants of βnBOR and αGB). The α phase usually exists as a strengthening phase in titanium alloys. Due to the different orientation relationships between the α and β phases, the difficulty of dislocations passing through the interface between the two phases varies, resulting in different strengthening effects and plasticity differences. Therefore, a deep understanding of the α phase variant selection in titanium alloys is of great significance for achieving the control of alloy mechanical properties.

The main objective of this study is to determine the microstructural evolution of the new titanium alloy-TB17 and the preferred selection of its α variants, during the cooling process from the β single-phase region at different cooling rates. This present study would provide a better understanding of the process and mechanism of β→α microstructural transformation in metastable titanium alloys and provide technical guidance for realizing precise microstructural tailoring of new metastable β alloys.

## 2. Materials and Experimental Section

### 2.1. Materials

**Figure 1:**
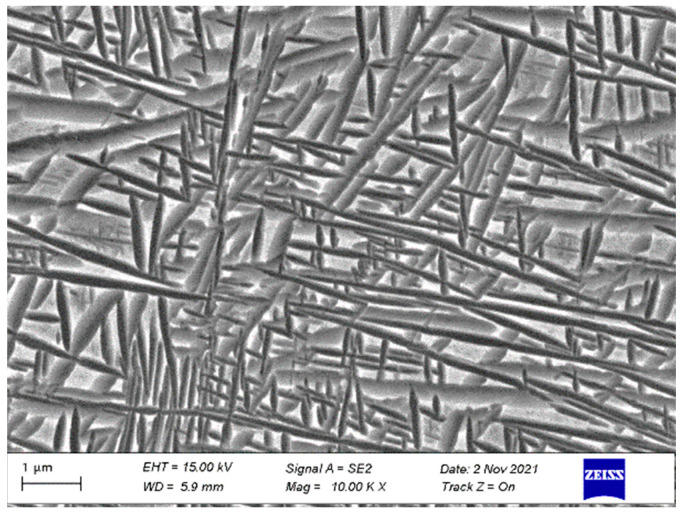
As-forged microstructure of TB17 titanium alloy.

The TB17 titanium alloy ingot was melted by multiple vacuum arc melting (VAR; Physcience Opto-electronics Co., Ltd., Beijing, China) three times to ensure uniformity of chemical composition, and then the open die was forged to 210 mm in diameter. The β→α transition temperature was 845 °C as measured by metallography. The chemical composition of TB17 titanium alloy is shown in Table 1 and the microstructure is shown in Figure 1. The microstructure is a typical basketweave structure. During the air cooling process, with a cooling rate of 15 °C/min after quasi-β forging, the lamellar α phase with different lengths is precipitated in the forging billet. The thickness of the lamellar α phase is between 50–100 nm, which is woven and evenly distributed on the matrix.

### 2.2. Heat Treatment

Cylindrical specimens of 3mm in diameter and 10 mm in length were machined from the TB17 titanium alloy forging stock using a wire-electrode cutting saw(Kunshan Ruijun Machinery Equit Co., Ltd., Kunshan, China) for continuous cooling tests at various cooling rates. To ensure the accuracy and representativeness of the experimental results, three samples were tested in each group of experiments. Various heat treatments with heating and cooling cycles were carried out using a thermal dilatometer (Linseis (Shanghai) Scientific Instrument Co., Ltd., Shanghai, China), which provides precise temperature control in a protective atmosphere. The specimens were initially heated to 900 °C and isothermally held for 30 min, then cooled continuously to room temperature at cooling rates of 1 °C/min, 10 °C/min, 15 °C/min, 20 °C/min, 50 °C/min, 100 °C/min, 150 °C/min, 200 °C/min, and 400 °C/min, respectively.

### 2.3. Microstructure Characterization

Microstructure observations were conducted on a *Sigma 300* scan electron microscope (SEM; Zeiss, Germany). The samples were polished with an automatic polishing machine (Trojan (Suzhou) Materials Technology Co., Ltd., Suzhou, China) and then etched in a corrosive solution (10% HF + 7% HNO_3_ + 83% H_2_O). Meanwhile, the microstructure was quantitatively investigated using Image-Pro Plus image 6.0 analysis software. The phase composition of the alloy at different cooling rates was determined by a D/Max 2500 X-ray diffractometer with a Cu Ka source (XRD; Rigaku, Tokyo, Japan). Moreover, the microstructure and crystallographic orientation of the α phase were observed using a JSM-7900F scanning electron microscope (SEM; Jeol, Tokyo, Japan) equipped with a Symmetry S2 electron backscatter diffraction detector (EBSD; Oxford Instrument Technology (Shanghai) Co., Ltd., Shanghai, China) and orientation imaging microscopy software Channel 5 (Oxford Instrument Technology (Shanghai) Co., Ltd., Shanghai, China). The microhardness of the alloy at different cooling rates was determined by the FMARS9000 Vickers hardness tester (FUTURE-TECH, Taiwan Nakazawa, Taiwan, China). 

## 3. Results and Discussion

### 3.1. Dilatometric Behaviors

**Figure 2:**
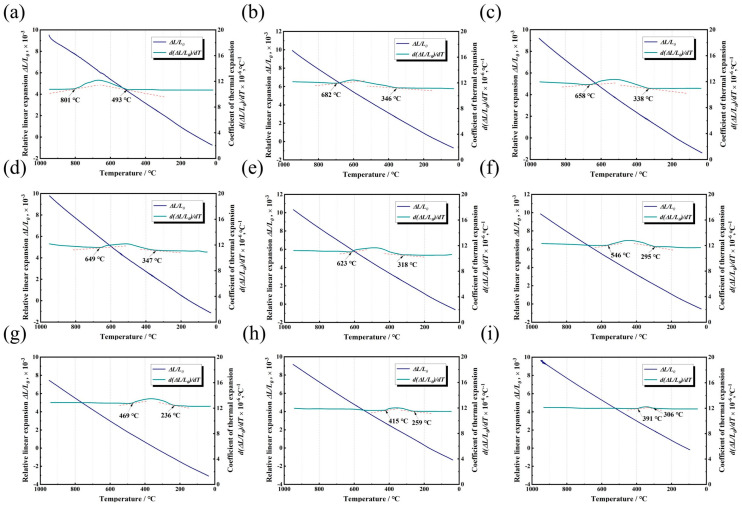
(**a**–**i**) are the relative coefficient curves and dilatometric curves of the alloy at cooling rates of 1 °C/min, 10 °C/min, 15 °C/min, 20 °C/min, 50 °C/min, 100 °C/min, 150 °C/min, 200 °C/min, and 400 °C/min, respectively; the horizontal solid lines in the figure are the relative coefficient curves, and the inclined solid lines are the dilatometric curves.

Figure 2 shows the relative expansion coefficient curves of TB17 titanium alloy at different cooling rates. As can be seen, the relative expansion coefficient at different cooling rates shows a parabolic curve change. During the cooling process, the relative expansion coefficient first maintains an approximate horizontal straight line. As the temperature decreases, the relative expansion coefficient increases first in a certain temperature range and then decreases slowly after reaching the peak until it drops to the initial relative expansion coefficient. The position corresponding to the temperature point where the relative expansion coefficient of the alloy changes significantly is taken at the beginning and end points of the phase transition. As can be seen from Figure 2, when the cooling rate is 1 °C/min, the phase transition start point and end point are 801 °C and 493 °C, respectively, and the relative expansion coefficient is the largest when the temperature is reduced to about 682 °C. As the cooling rate increases, the temperature range of the starting point and ending point of the phase transition gradually decreases. When the cooling rate increases to 400 °C/min, the start and end points of the phase transition are 391 °C and 306 °C, respectively, and the temperature corresponding to the maximum relative expansion coefficient is about 353 °C. In general, with the increasing cooling rate, the onset temperature of β→α phase transition of TB17 titanium alloy gradually decreases, while the phase transition final temperature first decreases and then increases. The dilatometric curves of TB17 titanium alloy at different cooling rates are shown in Figure 2. As the temperature decreases, the relative expansion at different cooling rates shows a linear downward trend, and there is no obvious deviation from the linear relationship. This is mainly because the volume increase of the alloy caused by the transition from β→α phase is about 0.17%, which is equivalent to the change of 0.06 ± 0.03% in the length direction [23,24]. It is difficult to show an obvious expansion or contraction effect on the relative expansion curve.

### 3.2. Establishment of CCT Curve of TB17 Titanium Alloy

**Figure 3:**
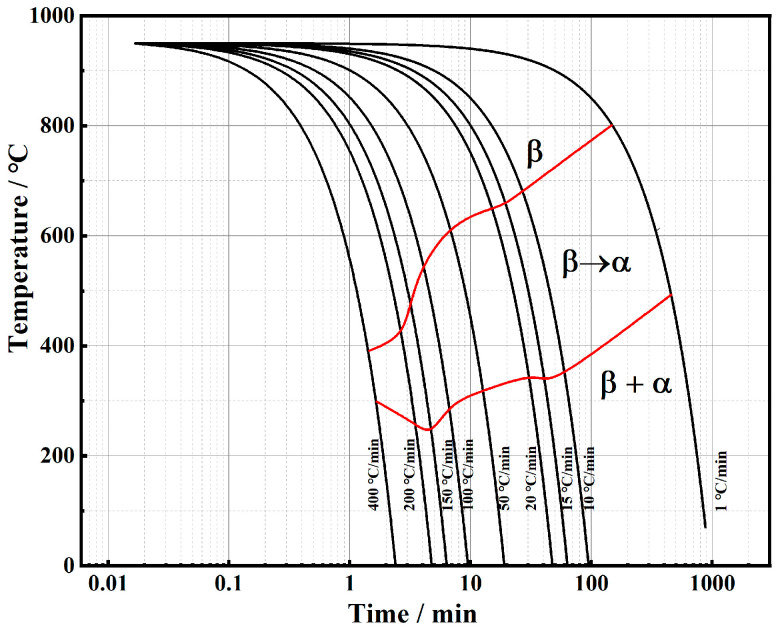
CCT diagram of TB17 titanium alloy.

The continuous cooling transformation (CCT) curve can reflect the phase transition law of the alloy during the cooling process. From this, the effect of the cooling rate on the start, end, and extent of the phase transition, as well as the structure of the transition can be obtained. As an effective foundation for analyzing the structure and properties of phase transformation products, it is of great guidance to formulate heat treatment process parameters and regulate the microstructure. According to the thermal expansion curves of TB17 titanium alloy at different cooling rates obtained in this chapter, the starting and ending temperature points of phase transformation at different cooling rates were obtained by the derivative method [25]. Combined with microstructure and microhardness analysis, the continuous cooling transformation kinetics curve (CCT curve) of TB17 titanium alloy was drawn, as shown in Figure 3. When the cooling rate is less than or equal to 400 °C/min, there is only α phase precipitation, and the precipitation of α phase mainly occurs in the middle and high-temperature region, no β→ω phase transition and β→α″ non-equilibrium phase transition occurs. The phase transition completes over a relatively wide range of temperatures and occurs at lower temperatures as the cooling rate increases. 

### 3.3. Microstructure Evolution

(1) XRD diffraction pattern of TB17 titanium alloy at different cooling rates

**Figure 4:**
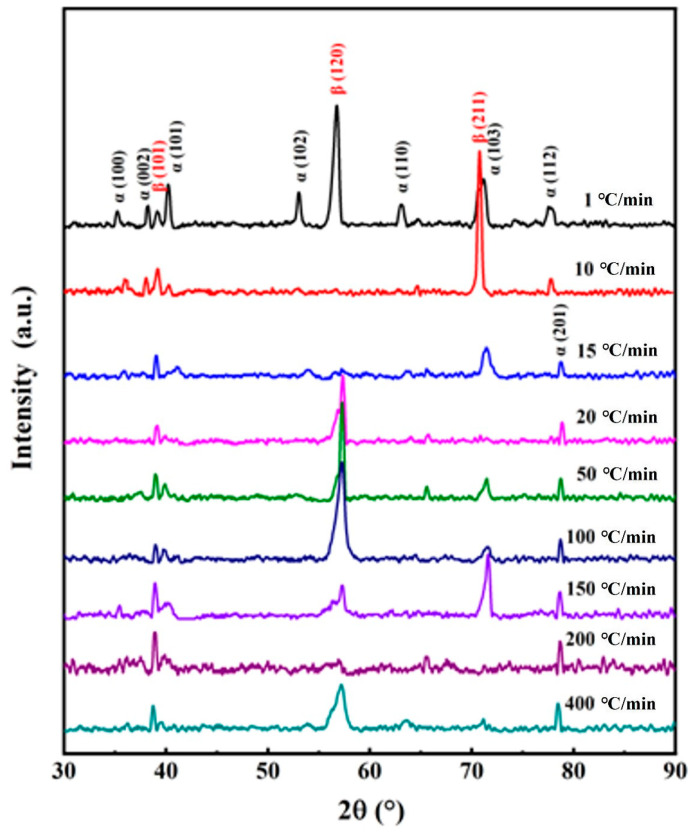
X-ray diffraction results of TB17 titanium alloy under different cooling conditions.

Figure 4 illustrates the XRD diffraction pattern of TB17 titanium alloy under different cooling conditions. When the cooling rate is fast (50 °C/min–400 °C/min), the phase composition of TB17 titanium alloy is not much different, mainly composed of β phase and α phase. no β→ω phase transition or β→α″ phase transition occurs. When the cooling rate is reduced to 50 °C/min, the relative intensity of the diffraction peaks of the α phase becomes stronger and their positions increase, especially the diffraction peaks of the α (103) phase are obviously enhanced, and the diffraction peaks of α (100), α (002) and α (101) appear simultaneously. This shows that as the cooling rate decreases, the content of α phase precipitated from the β matrix during cooling increases.

At a medium cooling rate (15 °C/min–20 °C/min), the phase composition of TB17 titanium alloy is mainly composed of β phase and α phase, and no β→ω phase transition or β→α″ phase transition occurs. As the cooling rate decreases, the relative intensity of the β (101) phase diffraction peak gradually decreases, and the relative intensity of the α (103) phase diffraction peak gradually increases. 

At a slow cooling rate (1 °C/min–10 °C/min), similarly to that of a high cooling rate, the phase composition of TB17 titanium alloy is mainly composed of β phase and α phase. As the cooling rate decreases, the relative intensity of the diffraction peak of the α (103) phase decreases, and the β (211) diffraction peak is differentiated, and strong β (120), α (102) and α (110) diffraction peaks appear. This shows that at a cooling rate of 1 °C/min, the α phase is fully precipitated in an approximately balanced phase transition.

(2) Microstructure characteristics of TB17 titanium alloy at different cooling rates

**Figure 5:**
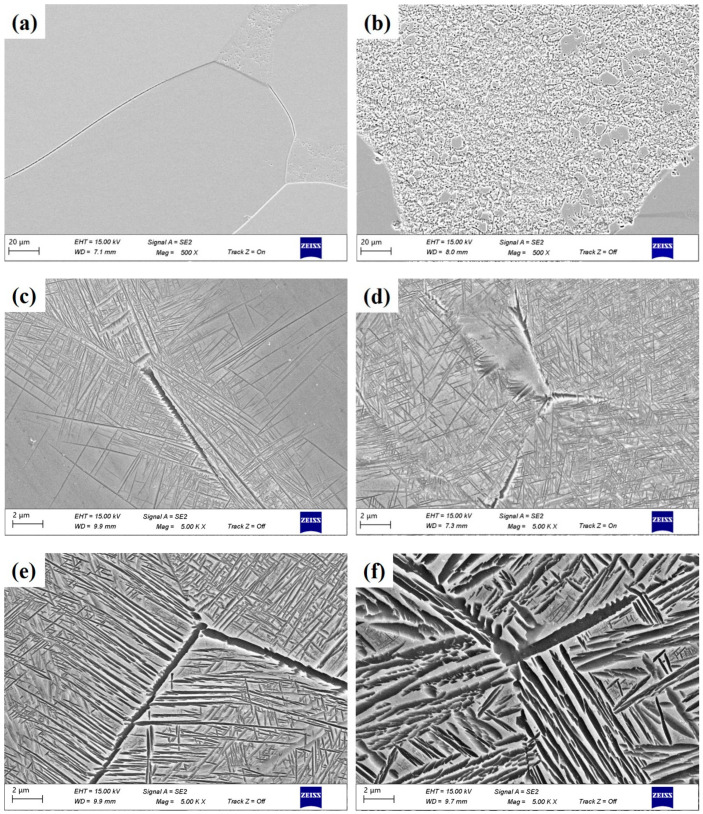
The back-scattered electron (BSE) images of TB17 titanium alloy at different cooling conditions. (**a**) 400 °C/min, (**b**) 50 °C/min, (**c**) 20 °C/min, (**d**) 15 °C/min, (**e**) 10 °C/min, (**f**) 1 °C/min.

Figure 5 reveals the microstructures of TB17 titanium alloy under different cooling conditions. When the cooling rate is 400 °C/min, due to the rapid cooling rate, a complete β grain is formed, and the grain boundary, a very small amount of α_WM_ is precipitated in the crystal, is obvious, as shown in Figure 5a. The size of α_WM_ is fine and grainy (or short rod-shaped with a small height–diameter ratio). As the cooling rate decreases, the number of α_WM_ precipitated from the β matrix gradually increases, the intragranular precipitation gradually changes to the simultaneous precipitation of intragranular and grain boundaries, and the size of its granular α_WM_ gradually becomes larger. However, the precipitation of the α_WM_ phase is not uniform, showing the phenomenon of selective precipitation in the local grain interior and grain boundary, which may be linked to the orientation difference between β grains. Figure 5b shows that when the cooling rate is reduced to 50 °C/min, bulk granular α-phases precipitate both within the grain and at the grain boundaries simultaneously. The number and size of the precipitated α-phases are significantly more than those of the faster cooling rate, which is in accordance with the XRD diffraction pattern (Figure 5).

The Burgers relation {0001}α∥{110}β is satisfied between α phase and β phase in titanium alloy, which means that there must be a semi-coherent relation and a non-coherent relation between α phase and β phase. Therefore, at the initial stage of nucleation, the α phase is granular or fine short rod-like structures. With the increase of precipitation time, the α phase migrates rapidly along the incoherent interface with the driving force of chemical potential energy difference and finally precipitates and grows into a lamellar or rod-like α phase. Due to the relatively fast cooling rate (50 °C/min–400 °C/min), the α phase has no time to grow into lamellar or rod-like, retaining the granular morphology of the α phase at the initial stage of nucleation.

At a cooling rate of 20 °C/min, α_GB_ precipitates at some grain boundaries, as shown in Figure 5c. Its thickness is about 0.1 μm–0.2 μm. At the same time, α_WGB_ and a small amount of α_WM_ are precipitated at the grain boundary. At this point, under rapid cooling conditions, the α phase has transformed from granular to fine lamellar. Similar to α_GB_, the distribution of α_WGB_ and α_WM_ is not uniform, which does not cover the whole grain boundary structure and does not occupy the whole β grain.

At a cooling rate of 15 °C/min, the precipitated α phase increases significantly, and the area covered by α_GB_ precipitation is more, as shown in Figure 5d. The α_WGB_ precipitates and connects closely with each other along the grain boundary, and keeps the approximate length and direction to grow continuously into the β grains, forming a large cluster domain. The α_WM_ in the crystal is densely distributed, but the precipitation orientation is dispersed, and the cross-distribution of each other is at the angle of 60°–90°. The thickness of the lamellar is about 15–30 nm. At this time, the precipitation of the α phase occupies almost the entire β grain, and the precipitation is sufficient.

In the condition of medium cooling rate (15 °C/min–20 °C/min), because of the reduced cooling rate, the α phase has a relatively sufficient time diffusion precipitation. When the temperature is reduced to below the phase transition point, the locally supersaturated metastable β phase is preferentially transformed under the action of rapid solute element diffusion, so the grain boundary and the vicinity of the grain boundary become the channel for rapid solute diffusion. The α/β interface migrates along the grain boundary under the control of solute diffusion, thus forming α_GB_ and fine lamellar α_WGB_. As the cooling rate decreases, more α_WGB_ precipitates along the grain boundary, the solute elements gradually diffuse into the crystal, and the fine lamellar α_WM_ is gradually precipitated from the inside of the β crystal, which is cross-distributed with α_WGB_.

Figure 5e shows that when the cooling rate is 10 °C/min, The α phase is fully precipitated, not only forming a complete α_GB_ but also α_WGB_ and α_WM_ are interlaced and distributed throughout the β grain, which is a typical Widmanstatten structure. Compared with the medium rate cooling condition, the α phase precipitated under the slow cooling condition is obviously wider, the lamellar thickness increases from 30–50 nm to 60–100 nm, and the grain boundary α phase is obvious, with a width of about 0.3 μm. Figure 5f shows that when the cooling rate is further reduced to 1 °C/min, due to the slow cooling rate, it can be clearly seen that large quantities of coarse lamellar α phases are precipitated on the β phase matrix, and the lamellar thickness is increased to 0.2 μm–0.5 μm. In the meantime, many fine α phases are precipitated around the coarse lamellar α phase, which are dotted or lamellar, distributed on the β matrix.

From the above analysis, it can be concluded that under the condition of slow cooling (1 °C/min–10 °C/min), the atomic diffusion is more sufficient, the migration of α/β interface during cooling is completely dominated by the diffusion of solute elements, and the α phase presents the process of precipitation → nucleation → growth. According to the Gibbs-Thomson effect, there is a concentration gradient in the β phase for α phase particles with different sizes, as shown by the fact that the smaller the particle, the higher the concentration of α stable elements around it. Therefore, the α-stable elements of the small-sized α phase continuously diffuse to the periphery of the large-sized α phase, which eventually leads to the gradual dissolution of the small-sized α phase and the continuous growth of the large-sized α phase. Therefore, as the cooling rate decreases, the α phase continues to grow, the α phase spacing gradually increases, and the macroscopic performance is the coarsening of the α phase.

In addition, α′, α″, and ω peaks are not observed in the XRD patterns of the TB17 titanium alloy regardless of the variation of the cooling rate. This suggests that there is only β→α diffusion transition as the phase transformation process during the continuous cooling process. In the α+β titanium alloy, with the increase in cooling rate, the content of α″ phase precipitated in the alloy increases and gradually transforms into orthorhombic martensite α″, such as TC21 [26]. Additionally, both α″ and ω phases can concurrently precipitate in Ti-1023 [27]. Generally speaking, the concentration of β stabilizing elements in the alloys determines their different precipitation mechanisms during cooling.

**Figure 6:**
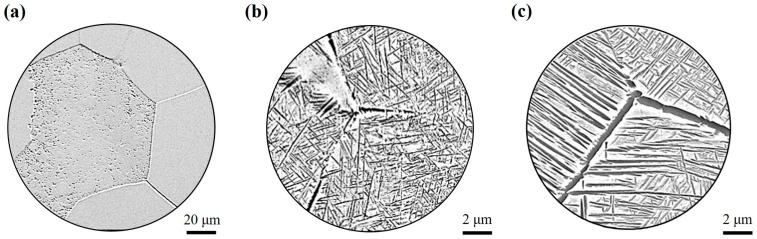
Evolution of microstructures under different cooling rates: (**a**) rapid cooling rate, (**b**) intermediate cooling rate, and (**c**) slow cooling rate.

For rapid cooling (50 °C/min–400 °C/min), a complete β grain is formed, and the grain boundary, a small amount of α_WM_ precipitated in the crystal, is obvious. At the medium cooling rate (15 °C/min–20 °C/min), α_GB_ is precipitated at some grain boundaries, along with a small amount of fine lamellar α_WM_, which are also precipitated at the grain boundaries. For slow cooling (1 °C/min–10 °C/min), the α phase is fully precipitated, and coarse lamellar α_WGB_ and α_WM_ are interlaced and distributed throughout the β grain. Finally, Figure 6 illustrates the precipitation processes of α_GB,_ α_WGB_, and α_WM_ at rapid, medium, and slow cooling rates, respectively.

**Figure 7:**
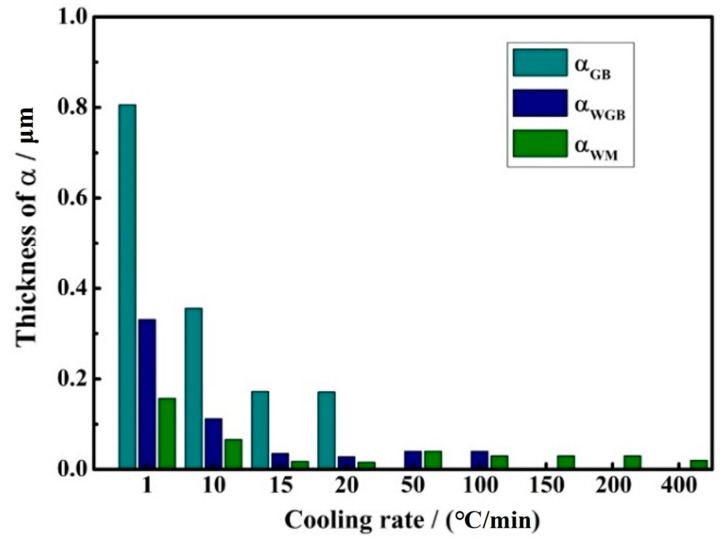
The thickness of α_GB_, α_WGB,_ and α_WM_ under different cooling rates.

Measurements and summaries of α phase widths are shown in Figure 7. The microstructure of TB17 titanium alloy varies greatly under different cooling rates. Combined with the SEM images of TB17 titanium alloy with different cooling conditions (Figure 5), it can clearly be seen that this difference is mainly reflected in the thickness of the secondary α phase and the morphology of the secondary α phase.

When the cooling rate is in the rapid cooling condition (150 °C/min–400 °C/min), a very small amount of granular α_WM_ is precipitated in the crystal. As the cooling rate decreases, the α_WM_ precipitated in the crystal gradually increases, but the grain size does not change much, and its size is mainly concentrated between 20–40 nm. When the cooling rate is reduced to 50 °C/min–100 °C/min, the secondary α phase is precipitated in the crystal and the grain boundary simultaneously, showing the phenomenon of selective precipitation in the local grain and the grain boundary.

When the cooling rate is moderate (15 °C/min–20 °C/min), the secondary α phase precipitated in the alloy changes from granular to fine needle-like, and simultaneously α_GB_, α_WGB,_ and α_WM_. The thickness of the secondary α phase is not much different. The thickness of α_GB_ is about 0.1 μm–0.2 μm, and the thickness of α_WGB_ and α_WM_ is about 15–30 nm. When the cooling rate is at a slow cooling condition (10 °C/min and 1 °C/min), the α_GB_, α_WGB_, and α_WM_ grow rapidly, especially the α_GB_, which proliferates from 0.356 μm at 10 °C/min to 0.806 μm. This demonstrates that the cooling rate significantly impacts the thickness of α_GB_, α_WGB_, and α_WM_.

### 3.4. Microhardness

**Figure 8:**
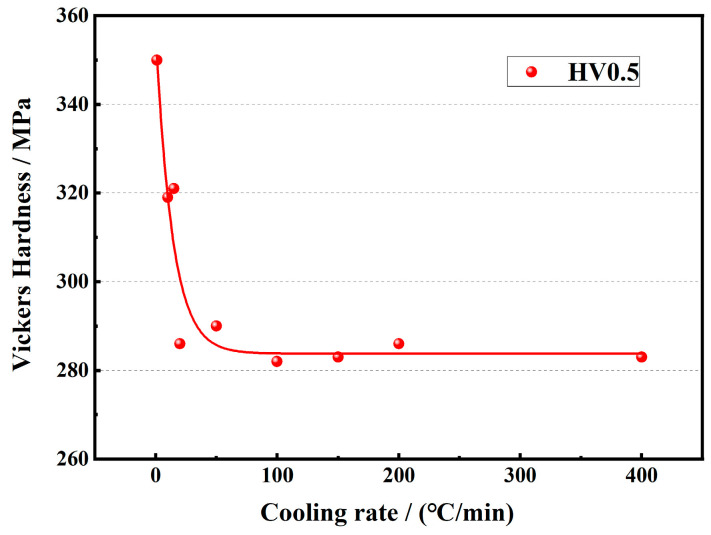
Microhardness curves of TB17 titanium alloy at different cooling rates.

The effect of the cooling rate on the microhardness of TB17 titanium alloy is shown in Figure 8. With the increase in cooling rate, the microhardness decreases rapidly. When the cooling rate is higher than 20 °C/min, the microhardness gradually becomes flat. This is because the TB17 titanium alloy has more alloying elements, and the faster cooling rate will inhibit the precipitation of the α phase, and the strengthening effect of the α phase cannot be obtained. Similar research results also appeared in the study of Zheng et al. [28]. The difference is that under slow cooling conditions, the α phase obtained in their study was finer and the microhardness improvement was more significant. In conjunction with the microstructure characteristics of Figure 5, the following conclusions can be drawn: When the cooling rate is a rapid cooling condition (50 °C/min–400 °C/min), the precipitated α phase is mainly granular, and its strengthening effect is limited; When the cooling rate is a medium cooling condition (15 °C/min–20 °C/min), the precipitated α phase is mainly fine lamellar, and the strengthening effect is obvious. Although the fine lamellar α sheet can rapidly increase the phase interface area and increase hardness, the increase in microhardness is not significant due to insufficient precipitation. When the cooling rate is a slow cooling condition (10 °C/min–1 °C/min), the precipitated α phase is mainly coarse lamellar, and the α phase is fully precipitated. The increase of α/β phase interface leads to an increase in the resistance of dislocation movement, and the microhardness is significantly improved.

### 3.5. Selection of α Variants

The microstructure of TB17 titanium alloy is a fully precipitated lamellar structure or basketweave structure, and the precipitated α phase has the phenomenon of variant selection behavior, that is, only several of the twelve α phase variants will be precipitated in the microstructure, and the precipitation of other-oriented α phases will be inhibited and will not be precipitated from the β matrix. In this paper, the complete cooling rate (1 °C/min) of α phase precipitation is used to study the selection of α phase precipitation variants.

**Figure 9:**
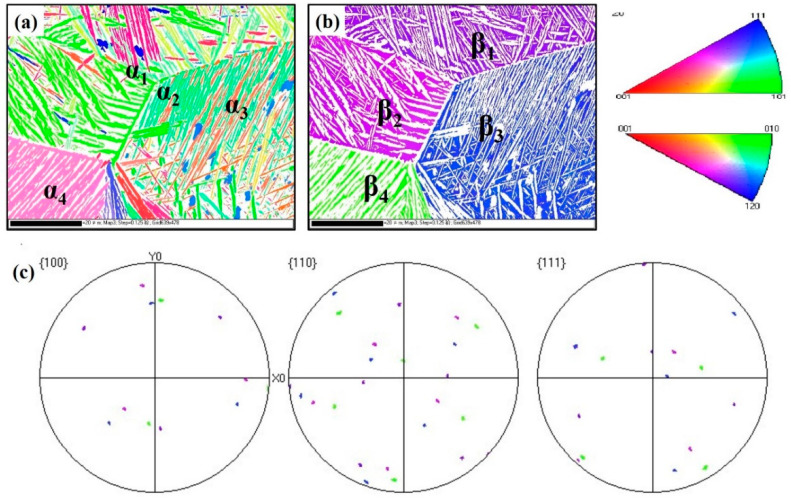
EBSD observation of specimen under the cooling rate of 1 °C/min. (**a**) IPF orientation distribution map of α phase, (**b**) IPF orientation distribution map of β phase, (**c**) corresponding pole Figure 9 of four β grains.

The EBSD observation of the microstructure under the cooling rate at 1 °C/min is shown in Figure 9. Figure 9a,b are the IPF orientation distribution maps of α phase and β phase, respectively. Their different colors mean different crystallographic orientations concerning the sample coordinate system. 

There are four β grains in the figure, and their positions on the pole figure are shown in Figure 9c. The micro-texture has formed with α_WGB_ precipitation at β/β grain boundary and there is no specific orientation relationship between adjacent β grains.

**Figure 10:**
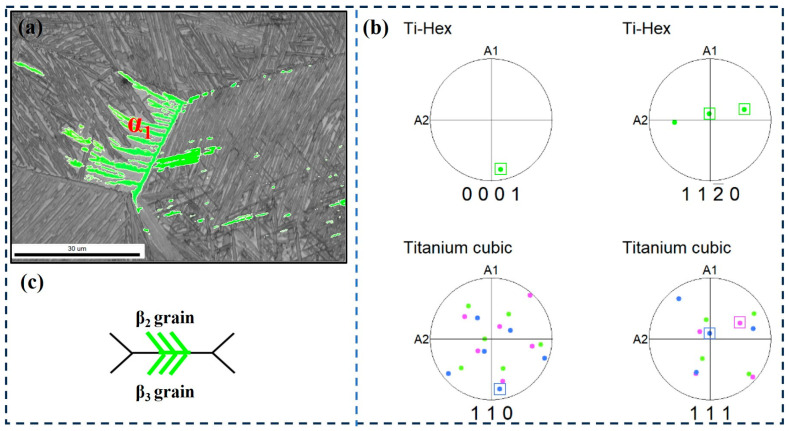
EBSD observation of specimen at the cooling rate of 1 °C/min. (**a**) Highlighted inverse pole figure map of α_1_; (**b**) Corresponding pole figures of α_1_, β_2_, and β_3_ grains; (**c**) Schematic illustration of the growth direction of α_1_.

**Figure 11:**
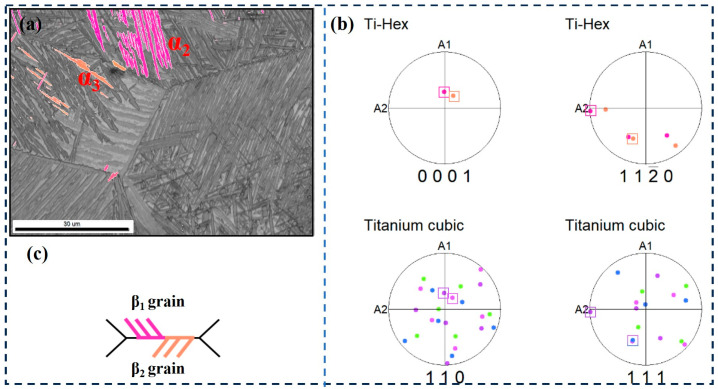
EBSD observation of specimens at the cooling rate of 1 °C/min. (**a**) Highlighted inverse pole figure map of α_2_ and α_3_; (**b**) Corresponding pole figures of α_2_, α_3_, β_2_, and β_3_ grains; (**c**) Schematic illustration of the growth direction of α_2_ and α_3_.

The selection of α variants near the original β grain boundary is mainly divided into three categories. The first type is that the α-clusters on both sides of the grain boundary have the same orientation, as shown in Figure 10. It can be seen that β_2_ and β_3_ have different orientations, but the α_1_ cluster on both sides of the β_2_ and β_3_ grain boundaries is consistent with the orientation of the grain boundary α (Figure 11c). Accordingly, Figure 10b shows the pole figure of α_1_ and β_2_, β_3_ grains on both sides of the grain boundary. The results show that α_1_, β_2_ and β_3_ grains satisfy the Burgers relationship, namely, {110}β_2_//{0001}α_1_, <111>β_2_//<11-20>α_1_ and {110}β_3_//{0001}α_1_, <111>β_3_//<11-20>α_1_. This indicates that the two clusters that maintain the Burgers relationship on either side of the adjacent β grains grow in different orientations but have the same crystallographic direction.

The second type is that the α-clusters on both sides of the grain boundary have different orientations, as shown in Figure 11. It can be seen that α_2_ grows from the β_1_ and β_2_ grain boundaries to the β_1_ crystal, and α_3_ grows from the β_1_ and β_2_ grain boundaries to the β_2_ crystal (Figure 11c), their c-axis angle is about 60°. Figure 11b is the pole figure of α_2_, α_3_ and β_1_, β_2_ grains. The {0001} pole figure and {11–20} pole figures of α_2_ correspond to the {110} pole figure and {111} pole figure of β_1_ grains, respectively, i.e., β_1_ is the Burgers grain of the cluster α_2_. In addition, the {0001} and {11–20} pole figures of α_3_ correspond to the {110} and {111} pole figures of β_2_ grains, respectively, i.e., β_2_ is the Burgers grains of cluster α_3_. This indicates that the two bundles that maintain BOR on both sides of the adjacent β grains have different growth directions and different crystallographic directions.

**Figure 12:**
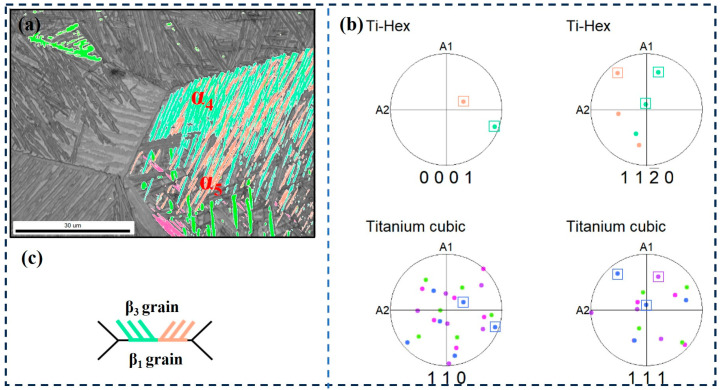
EBSD observation of specimen at the cooling rate of 1 °C/min. (**a**) Highlighted inverse pole figure map of α_4_ and α_5_; (**b**) Corresponding pole figures of α_4_, α_5_, β_1_, and β_3_ grains; (**c**) Schematic illustration of the growth direction of α_4_ and α_5_.

The third type is that the α clusters precipitated only in the β grains on one side of the grain boundary have different orientations, as shown in Figure 12. It can be seen that both α_4_ and α_5_ clusters grow along the grain boundaries of β_1_ and β_3_ to the interior of β_3_ grains; the growth directions are the same, but they have different orientations (Figure 12c). Figure 12b is the pole figure of α_4_, α_5_, β_1_, and β_3_ grains and shows that α_4_ and α_5_ only have a Burgers relationship with β_3_. That is to say, for clusters α_4_ and α_5_, β_3_ is a Burgers grain, and β_1_ is a non-Burges grain. This indicates that the α-bundles that maintain the Burgers relationship with only one side of the adjacent β grains may have the same growth direction, but different crystallographic directions, and these two directions correlate to two variants of the given {0001}α//{110}β.

To sum up, the cooling rate has a significant impact on the microstructure evolution and mechanical properties of the TB17 alloy. The change in cooling rate causes variations in the size and morphology of α_GB_, α_WGB_, and α_WM_ phases that are in a competitive state with each other. The increase in cooling rate leads to a decrease in the content of the α phase, which is the reason for the decrease in alloy strength. In addition, with changes in cooling rate, three different types of α variant selection near the original β grain boundary appeared in the alloy.

## 4. Conclusions

In this study, the microstructure evolution, microhardness, and crystallographic characteristics of typical α precipitates in the new metastable β titanium alloy TB17 under continuous cooling conditions at different rates have been thoroughly investigated. The conclusions are as follows:

(1) The continuous cooling transformation (CCT) diagram reveals when the cooling rate is less than or equal to 400 °C/min, only α phase precipitation occurs, without β→ω phase transition or β→α″ non-equilibrium phase transition.
(2) Not uniform of α_WM_ precipitated phase, a small amount of fine lamellar α_WGB_ precipitated phase formed under the low, intermediate, and high cooling rates, respectively. This is caused by the competitive growth of α_GB_, α_WGB_, and α_WM_.
(3) As the cooling rate increases, the formation of α phase precipitations is inhibited and the strengthening effect of α phase cannot be obtained, resulting in the microhardness decreasing rapidly. The precipitation of the coarse α phase during slow cooling results in a significant increase in microhardness.
(4) The selection of α variants near the original β grain boundary is mainly divided into three categories. The first type is that the α-clusters on both sides of the grain boundary maintain the BOR with either side of the adjacent β grains but grow in different directions. The second type is that the α-clusters on both sides of the grain boundary maintain BOR with one side of the adjacent β grains and grow in different directions. The third type is that the α clusters that precipitated only on one side of the grain boundary maintain the BOR with only one side of the adjacent β grains. It may have the same growth direction, but different crystallographic directions, and these two directions correspond to two variants of the given {0001}α//{110}β.

## Figures and Tables

**Table 1:** Chemical compositions of TB17 alloy used in this study (wt.%).

Element	Al	Mo	V	Nb	Cr	Zr	Sn	Fe	O	Ti
wt.%	3.91	6.84	1.19	2.05	2.71	1.02	1.02	0.04	0.089	Bal.

## Data Availability

All data generated or analyzed during this study are included in the present article.
